# Photochemical Synthesis and Versatile Functionalization Method of a Robust Porous Poly(ethylene glycol methacrylate-co-allyl methacrylate) Monolith Dedicated to Radiochemical Separation in a Centrifugal Microfluidic Platform

**DOI:** 10.3390/mi7030045

**Published:** 2016-03-10

**Authors:** Marion Losno, Ivan Ferrante, René Brennetot, Jérôme Varlet, Cécile Blanc, Bernard Grenut, Etienne Amblard, Stéphanie Descroix, Clarisse Mariet

**Affiliations:** 1 Den—Service d’Etudes Analytiques et de Réactivité des Surfaces (SEARS), CEA, Université Paris-Saclay, F-91191 Gif sur Yvette, France; marion.losno@cea.fr (M.L.); rene.brennetot@cea.fr (R.B.); jerome.varlet@cea.fr (J.V.); cecile.blanc@cea.fr (C.B.); 2 MMBM Group, Institut Curie Research Center, CNRS UMR 168, F-75005 Paris, France; ivan.ferrante@curie.fr (I.F.); stephanie.descroix@curie.fr (S.D.); 3 Den—Service d’Etude du Comportement des Radionucléides (SECR), CEA, Université Paris-Saclay, F-91191 Gif sur Yvette, France; bernard.grenut@cea.fr (B.G.); etienne.amblard@cea.fr (E.A.)

**Keywords:** nitric acid, radiochemistry, monolith, thiol-ene, click-chemistry, chromatography, anion-exchanger, nuclear spent fuel

## Abstract

The use of a centrifugal microfluidic platform is an alternative to classical chromatographic procedures for radiochemistry. An ion-exchange support with respect to the *in situ* light-addressable process of elaboration is specifically designed to be incorporated as a radiochemical sample preparation module in centrifugal microsystem devices. This paper presents a systematic study of the synthesis of the polymeric porous monolith poly(ethylene glycol methacrylate-co-allyl methacrylate) used as a solid-phase support and the versatile and robust photografting process of the monolith based on thiol-ene click chemistry. The polymerization reaction is investigated, varying the formulation of the polymerisable mixture. The robustness of the stationary phase was tested in concentrated nitric acid. Thanks to their unique “easy-to-use” features, centrifugal microfluidic platforms are potential successful candidates for the downscaling of chromatographic separation of radioactive samples (automation, multiplexing, easy integration in glove-boxes environment, and low cost of maintenance).

## 1. Introduction

The precise and accurate chemical analysis of nuclear spent fuel (NSF) represents a critical part of materials control and accountancy and plays an essential role in designing future nuclear fuel cycles, with regard to reprocessing as well as for waste management. The analysis of each radionuclide remains difficult because NSF samples exhibit an extreme chemical diversity stemming from several schemes of neutron capture, fissions, or activation reactions that occur in reactors. This leads to the formation of the so-called transuranium radionuclides (Np, Pu, Am, Cm) as well as a wide variety of fission products (FP) constituted of lanthanides like Eu. After their dissolution in nitric acid (HNO_3_) their concentration ranges usually from a few pg·L^−1^ for some fission products up to g·L^−1^ level for uranium, while plutonium concentration depends on the type of fuel and ranges from 0.1 to 10 mg·L^−1^. Moreover, after a prolonged stay in a nuclear reactor, NSF samples are very “hot” products and their analyses require an appropriate shielding level to reduce the dose uptake for the analyst, such as remote handling in hot cells, or extreme dilution.

Analysis of radionuclides present in these samples is carried out according to procedures including the sequence of several separation/purification operations before performing the measurement by liquid scintillation spectrometry gamma, X-ray spectrometry, and mass spectrometric techniques. These operations, based on the techniques of chromatography, liquid-liquid extraction, and precipitation/dissolution [1], are primarily manual and involve the presence of an operator. They have a large number of specific constraints linked to the sample (radiation protection) and to the accuracy required for the results (cross contamination). This is why the design of miniaturized, easy to use, disposable, and automatable analysis tools was studied. The use of such tools would, indeed, reduce the overall time of analysis but also decrease consumption of radioactive samples. It implies the simplification of radiation protection [2,3] and the production of waste at the end of the analytical cycle [4].

Centrifugal microfluidic platforms are attractive candidates to carry out the chromatographic separation step involved in the chemical analysis of NSF samples because fluid handling is “simply” provided by the use of the centrifugal force while no connection to external pumps or power supplies is needed. This simplifies tremendously the installation and maintenance for procedures to be implemented in a highly radioactive area such as glove boxes. The field of centrifugal microfluidics has experienced significant growth in the past two decades, especially in the development of fluidic technologies suitable for sample-to-answer devices for *in vitro* diagnostics [5,6,7]. These technologies led to the creation of completely automated and multiplexed centrifugal devices, known as Lab-on-CD (LoCD) [8,9,10] and allow benefits in terms of reproducibility and time of analysis. In the LoCD, specific forces control fluids. Inertial forces (centrifugal force, Coriolis force, and Euler force) induced by rotating the CD are utilized to move the liquids from the rotating center to the periphery, and transport samples and reagents from the inlet reservoirs to the metering, mixing, reaction, detection, and waste reservoirs [11,12]. Solid phase extraction and/or chromatographic separation in LoCD deserve special considerations because both require the integration, in the device, of a stationary phase which selectively retains targeted analytes.

**Figure 1:**
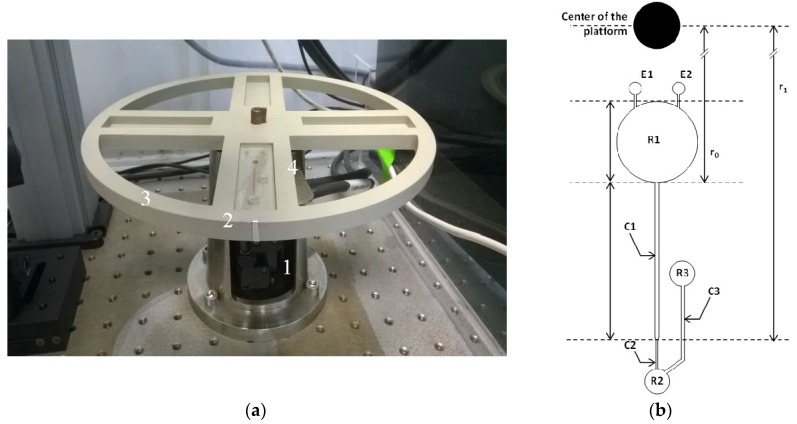
(**a**) Centrifugal microfluidic platform including: **1** the rotor securely affixed onto **2** an aluminum chuck, **3** the machined PEEK (PolyEtherEtherKetone) holding platform, and **4** the inserted microchip; (**b**) Design of the microchips (7 cm × 2.2 cm): **C1** ion-exchange column, **C2** restriction channel, **C3** vent channel, **R1** injection reservoir, **R2** collection reservoir and chromatographic connection, **R3** vent and chromatographic connection, **E1** entry port, and **E2** vent port of the injection reservoir, from [15].

Only few studies have recently reported the integration of a monolith in a centrifugal microfluidic platform for elemental analysis [13,14,15]. The Penrose team [16] has developed a centrifugal device combining a chromatographic column and microfluidics functions initially for the separation of dyes, then Saline *et al.* [14] have applied it to the analysis of V, Pb, Ni, Cu and Co in the water by Inductively Coupled Plasma Mass Spectrometry (ICP-MS) coupled with laser ablation. In a previous study [15], a chromatographic separation of uranium and europium in concentrated hydrochloric acid by the means of a microsystem implanted on a centrifugal platform was developed (Figure 1a). The platform can hold four thermoformed COC (Cyclic Olefin Copolymer) microchips at a time. The centrifuge motion of the platform was driven by a servo motor (Yaskawa SGMAV 01ADA61, Yaskawa America, Inc., Waukegan, IL, USA) with a corresponding motor controller (Servopack SGDV R90A01A, Yaskawa America, Inc., Waukegan, IL, USA). This work demonstrated the potentialities of centrifugal microfluidic platforms as a viable alternative to the standard procedure for chromatographic anion-exchange separations with high extraction yield for U and Eu. However, this separation was achieved in a hydrochloric acid medium whereas the established medium for the separation of radionuclides is concentrated nitric acid. The anion exchange monolithic stationary phase was not compatible with the concentrated nitric medium necessary for the separation of uranium and europium because the function with the anion exchanger group was deteriorated.

The aim here is to develop a new versatile functionalization method of a monolithic stationary phase compatible with concentrated nitric acid that could be integrated into the previous developed centrifugal microsystem (Figure 1b). The stationary phase has to present a high chemical stability and be easily synthesized and functionalized in a microsystem. So, first, we developed a photo-polymerized polymethacrylate monolith. Second, we studied a thiol-ene click-reaction chosen as a versatile photo-functionalization method. Then the robustness of the functionalized stationary phase was tested in [HNO_3_] > 5 mol·L^−1^.

## 2. Materials and Methods

### 2.1. Materials

**Figure 2:**
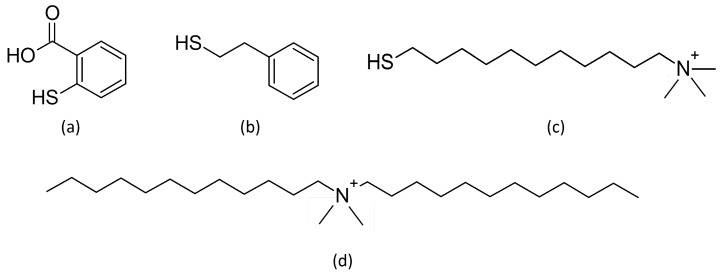
(**a**) Thiosalicylic acid; (**b**) 2-phenylethanethiol; (**c**) (11-Mercaptoundecyl)-*N*,*N*,*N*-trimethylammonium; (**d**) didodecyldimethylammonium.

Ethylene glycol methacrylate (97%, EDMA), allyl methacrylate (98%, AMA), 2,2-dimethoxy-2-phenylacetophenone (>99%, DMPA), 1,4-butanediol (99%), 1-propanol (99.7%), methanol (HPLC-grade), thiosalicylic acid (Figure 2a), (97%), 2-phenylethanethiol (98%) (Figure 2b), (11-Mercaptoundecyl)-*N*,*N*,*N*-trimethylammonium (≥90%, ammonium thiol) (Figure 2c), didodecyldimethylammonium bromide (98%), and HNO_3_ 65% (wt) were purchased from Sigma Aldrich (Isle-D’Abeau, France). All aqueous solutions were prepared using >18 MΩ deionized water (Direct-Q UV3, Millipore, Billerica, MA, USA).

The illumination system used for photopolymerization reactions was a Bio-link BLX cross-linker (VWR International, Strasbourg, France) equipped with five 8 W UV tubes emitting at 365 nm. A UVX-radiometer (Fisher Scientific, Lyon, France) equipped with a 365-nm sensor (1 cm^2^) was used to record the total UV-light energy supplied during each polymerization. Scanning electron microscopy (SEM) images were acquired using a JEOL JSM 7000F Scanning Electron Microscope (Jeol, Croissy-sur-Seine, France) after metallization of the monoliths. A Micromeritics’ AutoPore IV 9500 Series (Micromeritics Instrument Corporation, Norcross, GA, USA) was used to characterize the monolith’s porosity by applying various levels of pressure to a sample immersed in mercury. The pressure required to intrude mercury into the sample’s pores is inversely proportional to the size of the pores. The infrared spectrophotometer used for the characterization by Fourier transform infrared spectroscopy with attenuated total reflectance (FTIR-ATR) of functionalization is a Bruker Equinox 55S (Bruker Corporation, Coventry, UK) with a Golden GateTMSpecac (Specac, Orpington, UK) and a DTGS (deuterated triglycerin sulfate) detector. Air is measured as the background signal. Spectrum is acquired between 5800 and 550 cm^−1^ with a 2 cm^−1^ resolution, 32 scans through OPUS software. The functionalized monolith is simply put on the germanium crystal without any preparation of the sample. Mass spectrometry analyses are performed in positive ion mode using a Q-TOF II mass spectrometer (Micromass, Manchester, UK). Ions are recorded between ratios *m*/*z* (ion mass/ion charge) 50 and 1000. All the analytical parameters remain constant: sample cone voltage, 30 V; capillary voltage, 3 kV; source temperature, 100 °C; and desolvation temperature, 150 °C. Instrument control and data acquisition are performed using MassLynx 4.1 software within 1 min. A blank is recorded during 1 min before each sample analysis.

### 2.2. Synthesis of the Poly(ethylene glycol methacrylate methacrylate-co-allyl methacrylate) Monolith (also Referred to as Poly(EDMA-co-AMA) Monolith)

A monomer mixture (EDMA, AMA, DMPA) was mixed with a ternary porogen of 1,4-butanediol, 1-propanol, water, in a glass test beaker of 1 mL. Each compound mixture (1,4-butanediol, 1-propanol, water, EDMA, AMA) is weighed successively, in order, in a 1 mL glass beaker. 1 wt % with respect to the monomers of 2,2-dimethoxy-2-phenylacetophenone was added to the mixture as a polymerization initiator. Prior to irradiation, the mixture is set to shake for 10 min, then covered with parafilm to undergo the degassing of dissolved oxygen in solution by sonication for 7 min and placed for under 5 min in nitrogen flux [17]. The photopolymerization reaction was then initiated by a 365 nm UV light for various irradiation times with a power of 2.85 mW·cm^−2^. During the irradiation step, the temperature increased slightly in the UV-oven (a few degrees), without any impact on the final monolith properties compared with temperature controlled photopolymerization. The resulting monolith was then removed from the test tube and thoroughly washed with methanol.

### 2.3. Functionalization of the Monolith

Functionalization of the methacrylate monolith was carried out with 10 or 20 mg of monolith in a constant 1 mL solution. A fresh 0.65 mol·L^−1^ thiol stock solution is prepared before each set of experiments. The concentration of the 1 mL reaction mixture is then adjusted from the stock solution.

Monolith, stock thiol solution, methanol and DMPA are added one by one. The resulting mixture is stirred for a couple of minutes before it is put under the 365 nm UV-light. It is then diluted by 1 mL methanol before the supernatant is eliminated. The resulting monolith is then washed by 1 mL methanol according to the following procedure: 10 min stirring, 20 min centrifugation (10,000 rpm), elimination of the supernatant liquid, drying.

## 3. Results and Discussion

**Figure 3:**
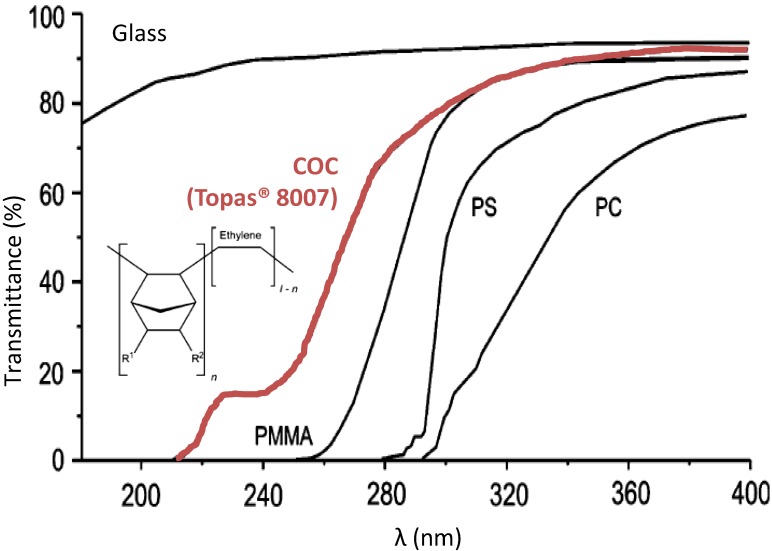
Comparison of the optical transmission of the COC (Topas^®^-8007, TOPAS Advanced Polymers, Frankfurt-Höchst, Germany) with other thermoplastics commonly used in microfluidics taking glass as Reference [18] (chemical Structure of the Topas COC), sheets of 2 mm thicknesses. PMMA: polymethylmethacrylate, PS: polystyrene, PC: polycarbonate.

Photochemistry is a simple tool to integrate and functionalize phase stationary *in situ* in a microchannel. However, this requires that the material used for the microfabrication of the microsystem is compatible with the wavelength used for the photochemistry. The COC has excellent chemical resistance to many solvents as well as strong acids, a high thermal and mechanical resistance, and optical properties exceeding those of other thermoplastics with a quasi-UV transparency up to 300 nm (Figure 3—red line) [18].

### 3.1. Monomers Choice and Optimization of the Monolith Synthesis

**Figure 4:**
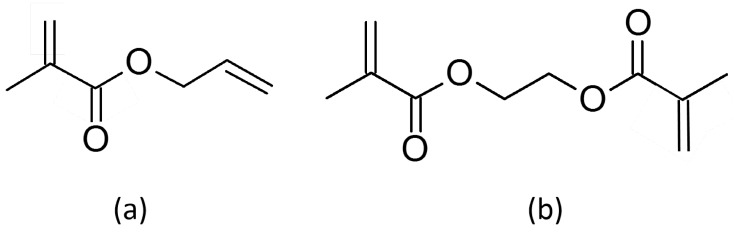
Monomers used for the copolymerization of the monolith (**a**) functional AMA and (**b**) crosslinker EDMA.

Since their introduction in the early 1990s [19], polymethacrylate monoliths have emerged as a powerful alternative tool in chromatographic column technology. Methacrylate polymers are widely used in very different fields such as separation of benzene derivatives [20,21,22], peptides [21], or proteins [23]. They are also used to separate small molecules [24] or atoms in hard medium as shown in a previous work [15]. The combination of their singular porous properties, the various chemistries available and their relatively simple implementation in columns with small internal diameters make them particularly attractive for the new chromatographic challenges of complex matrices analysis [25] or on-chip separations chromatography [26]. Moreover, methacrylate polymerize under UV radiation at 365 nm, *i.e.*, at a wavelength that is compatible with the use of the COC [25]. This widely used way of polymerization allows a localization of the polymerized area thanks to the use of a mask [27,28]. The monolith is composed of two monomers: the crosslinker provides the polymeric network and the functional monomer leads the surface reactivity. So the monolith could be adapted to the functionalization method and the analytical separation needed by a simple change in the monomer mixture. EDMA (Figure 4b) is a currently used crosslinker [20,22,29] because of its stability and ability to create a homogeneous network with other methacrylate monomers [30]. EDMA was chosen as crosslinker in this study. The functional monomer AMA (Figure 4a) was selected because of the interesting reactivity of the carbon double bond which enables a wide scope of chemical reactions especially in the field of click-chemistry.

To initiate the photopolymerization, an initiator is necessary and 2,2-dimethoxy-2-phenylacetophenone (DMPA) was chosen because of its good efficiency compared to others, such as benzophenone [31]. If the choice of the monomers and the initiator is important, the solvent of the polymerization mixture, the porogen, has a large influence on the monolithic structure [32,33]. Its aim is to create micro- and macropores during the reaction to allow the circulation of the mobile phase and good interaction with the analytes during the separation process. As the objective is to perform a monolith with a similar structure in terms of porosity, pores size, and globules size than that obtained previously [15] but resistant in concentrated nitric acid, the ternary mixture water/1,4-butanediol/1-propanol was kept [34]. Water is added as a porogen because of its effect for the separation of small analytes such as rare earth [35], metals [35,36,37], or U, Eu [15]. Time, ratio between the two monomers, and ratio between the constituents of the porogen were optimized. Scanning Electron Microscopy (SEM) was used to characterize pores and globules size and Mercury Intrusion Porosimetry (MIP) to get the porosity measurement. A longer reaction time, an increase of proportions of EDMA, or the addition of water to the porogenic mixture induces larger globules. Several experimental conditions were tested to reach the target values from previous studies [15].

**Figure 5:**
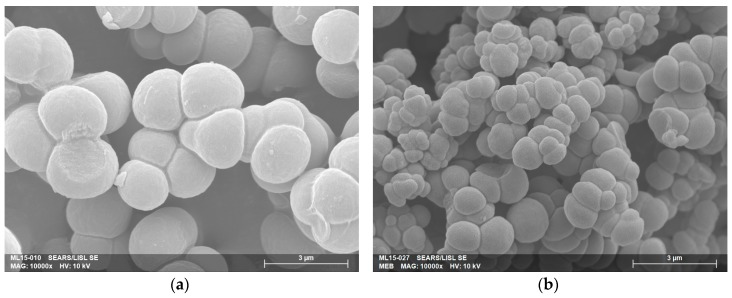
MEB pictures (×10,000) of two monoliths synthetized with different experimental conditions. (**a**) Monolith a: 7 wt % water, 18 wt % 1,4-butanediol, 35 wt % 1-propanol, 10 wt % EDMA, 30 wt % AMA, 1 wt % DMPA, polymerization time 13 min 20 s; (**b**) Monolith b: 1 wt % water, 24 wt % 1,4-butanediol, 35 wt % 1-propanol, 24 wt % EDMA, 16 wt % AMA, 1 wt % DMPA, polymerization time 10 min.

Figure 5 shows the influence of experimental conditions on the morphology of the monolith, especially the globule size. The monolith presented in Figure 5a is composed of nodules with a size of 2.01 ± 0.27 µm, whereas the nodule size is half (1.02 ± 0.83 µm) for the second experimental conditions (Figure 5b). To obtain the globule size, SEM pictures were analyzed manually through the ImageJ software [38] which could explain the high error for the pore size measurement. Indeed, globules were assumed to be perfect spherical objects and 2D measurements were extrapolated. Table 1 presents the results of the characterizations of the monoliths a and b. Monolith b is the monolith with the nearest structure of that obtained previously. Concerning the total porosity measurement the change of functional monomer could explain the differences between the new and previous monoliths.

The synthetized polymethacrylate structure is close to the target one and the size of the globules allows functionalization as proved in the previous work [15].

### 3.2. Choice and Optimization of a Robust and Versatile Functionalization Method of the Monolith

Functionalization of the monolith is an indispensable step to create the desired affinity of the stationary phase for separation of radionuclides in nitric acid. As explained in the previous part, AMA was chosen as the functional monomer because of its C=C double bond extremity that allows the use of a wide scope of reactions including click-chemistry. Click-chemistry was first studied by Shapless *et al.* in the 90s [39] and offers several advantages like stereospecificity, high yield, simplicity of experimental conditions, easy purification (if needed), or mild solvent. Click-chemistry often involves C=C double bond but only two are mainly used in literature for surface modification [40]: azide-alkyne cycloaddition and thiol-ene reaction. Azide-alkyne cycloaddition is a copper-catalyzed reaction which means that copper could be present in the monolith even after it was washed. It could create a chemical interference in the analytical procedure. This is the reason why thiol-ene reaction was preferred for functionalization. Furthermore, the thiol-ene click-reaction can be performed by photochemistry [41] and allows the localization of the functionalization [42,43].

The aim of the study is to create a strong covalent bond between monolith and the functionalization molecule to obtain a final structure resistant to [HNO_3_] = 8 mol·L^−1^. First, robustness of the created C–S bond was experienced through two aromatic compounds. Thiosalicylic acid (Figure 2a) and 2-phenylethanethiol (Figure 2b) were chosen because they own an aromatic cycle that can be easily characterized by spectroscopic methods. Unlike the 2-phenylethanethiol, the aromatic cycle is conjugated with the thiol function in the thiosalicylic acid. This difference could affect the stability of the C–S bond. Since the complexation of the uranyl UO_2_^2+^ by nitrate in concentrated HNO_3_ leads to the formation of complexes of the form [UO_2_(NO_3_)_*x*_]^(2−*x*)^ (1 ≤ *x* ≤ 3), anion exchange chromatography is commonly used to separate uranium (VI) from other radionuclides [43,44,45,46,47]. Anionic complexes of U are formed in nitric acid for a concentration exceeding 4 mol·L^−1^ [47] whereas Eu(III) formed cationic complexes. Thus, the functionalization by an anion exchanger will enable the separation of the two components in [HNO_3_] > 4 mol·L^−1^. In a first step U(VI) will be fixed in the stationary phase and the Eu(III) will be eluted. Then U(VI) will be eluted in diluted HNO_3_. Strong anion exchange resins used contain quaternary ammonium groups [44,45,46,47]. So, a thiol functionalized with a quaternary ammonium, the commercially available (11-Mercaptoundecyl)-*N*,*N*,*N*-trimethylammonium (Figure 2c), was photografted to the stationary phase. The trimethyl ammonium function is expected to have a strong affinity for the anionic uranyl complexes.

**Figure 6:**
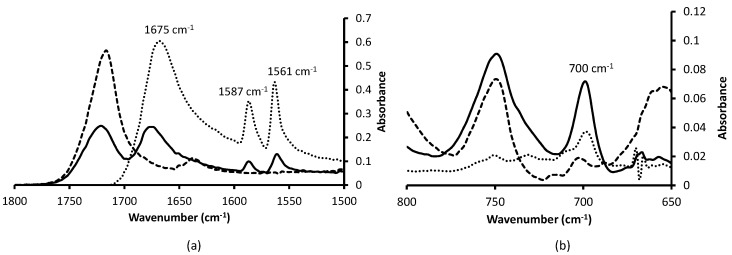
FTIR-ATR spectra of functionalized monolith focused on bands of interest. (**a**) Thiosalicylic acid; (**b**) 2-phenylethanethiol. Dotted line: thiol; dashed line: monolith before functionalization; solid line: monolith after functionalization.

The thiol-ene click reaction is well-known for its versatility [41] so the optimization of the reaction was carried out with thiosalicylic acid and confirmed with 2-phenylethanethiol because their IR characterization is easier. Various irradiation times and thiol/AMA ratios were tested and the optimal functionalization was obtained for 12.5 equivalents of thiol and 40 min under UV-light. FTIR-ATR was used to characterize the surface of the photografted monolith. Figure 6 a shows the infrared spectra and the characteristic bands of the grafted monolith by thiosalicylic acid: 1675, 1587 and 1561 cm^−1^ bands are specifics for thiosalicylic acid in the aromatic C=C zone [48,49]. Once the optimal experimental conditions were determined with thiosalicylic acid, the functionalization was performed with 2-phenylethanethiol (Figure 2b) for validating experimental conditions and the versatility of the method. The 700 cm^−1^ band (Figure 6b) is a characteristic band of aromatic C–H “oop” (out of plane) vibration [48,49] of the 2-phenylethanethiol.

**Figure 7:**
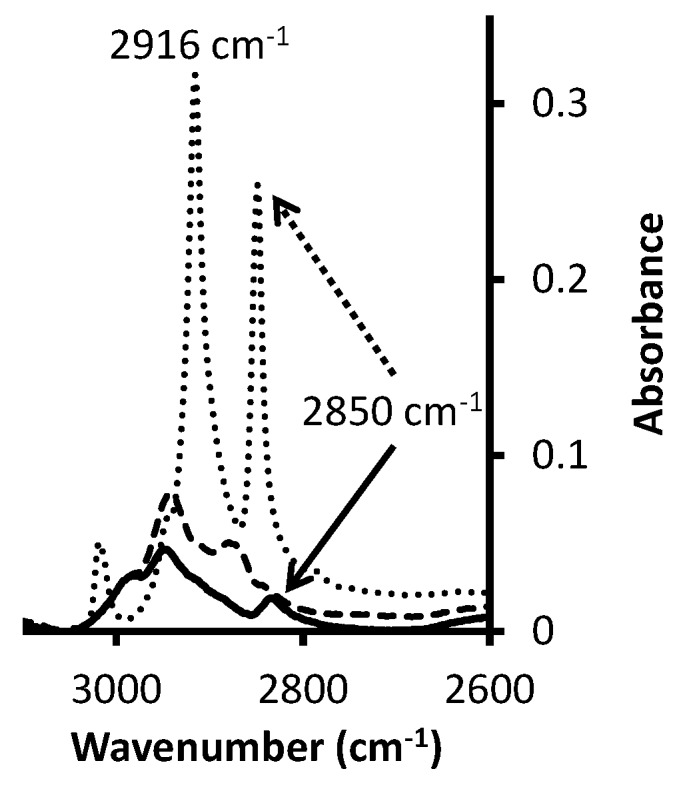
FTIR-ATR spectrum of (11-Mercaptoundecyl)-*N*,*N*,*N*-trimethylammonium functionalized monolith focused on bands of interest. Dotted line: thiol; dashed line: monolith before functionalization; solid line: monolith after functionalization.

Ammonium thiol was then grafted as can be seen in Figure 7. 2916 and 2850 cm^−1^ are characteristic bands of –CH stretch vibrations of –CH_2_– groups [48,49] of the (11-Mercaptoundecyl)-*N*,*N*,*N*-trimethylammonium molecule.

Infrared characterization allowed confirming the presence of an effective functionalization at the surface of the polymethacrylate monolith for three different functionalized thiols. Then, it can be concluded that the “click-chemistry” developed protocol is versatile.

### 3.3. Robustness in Nitric Acid Medium

**Figure 8:**
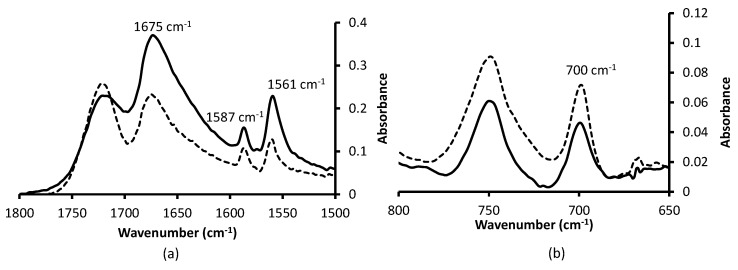
FTIR-ATR spectra of functionalized monolith focused on bands of interest. (**a**) Thiosalicylic acid; (**b**) 2-phenylethanethiol; dashed line: fresh functionalized monolith; solid line: functionalized monolith after 24 h in contact with [HNO_3_] = 8 mol·L^−1^.

For radiochemical protocols, the robustness of the C–S bond of grafted monoliths has to be experienced in the harsh separation medium. The robustness of the three functionalized monoliths was tested in [HNO_3_] = 8 mol·L^−1^. Functionalization by thiosalicylic acid and 2-phenylethanethiol are still visible on infrared spectra after 24 h of immersion in the acidic medium as shown Figure 8a,b, respectively. Either for a conjugated or a non-conjugated thiol, the C–S bond is resistant to an 8 mol·L^−1^ nitric acid solution.

As regards the (11-Mercaptoundecyl)-*N*,*N*,*N*-trimethylammonium, first results showed the disappearance of the signal of CH_2_ group of the aliphatic chains which means a degradation of the grafted molecule. Further experiments are then carried out to show the effective degradation of ammonium thiol in [HNO_3_] = 8 mol·L^−1^. Aliphatic chains present in the studied ammonium are assumed to be too short to compensate for the positive charge of the ammonium under such hard conditions. In fact, a mixture of ammonium with C8 and C10 chains (with C8 predominating) is commonly used in radiochemical procedures as a strong ammonium anion exchanger [44,45,46,47,50]. Therefore, the resistance to the acid of two ammonium molecules is then tested to confirm the influence of the aliphatic chains: the (11-Mercaptoundecyl)-*N*,*N*,*N*-trimethylammonium containing three methyl groups and one C11 chain linked to the nitrogen; and didodecyldimethylammonium (Figure 2d) composed of two methyl groups and two C12 chains bonded to the nitrogen. Each molecule was put for 24 h in [HNO_3_] = 8 mol·L^−1^ and analyzed by mass spectrometry.

**Figure 9:**
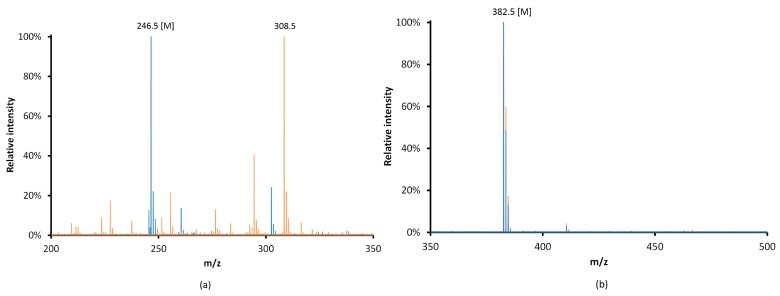
Mass spectra of M (**a**) (11-Mercaptoundecyl)-*N*,*N*,*N*-trimethylammonium and (**b**) didodecyldimethylammonium after contact with [HNO_3_] = 8 mol·L^−1^; blue line: *t*_contact_ = 0; orange line: *t*_contact_ = 24 h.

In Figure 9a, a modification of the mass spectrum of the (11-Mercaptoundecyl)-*N*,*N*,*N*-trimethylammonium immerged in nitric acid during 24 h is observed. The *m*/*z* 246.5 peak corresponding to the initial organic ion disappeared and gave way predominantly to a *m*/*z* 308.5 peak and a multitude of new ions. In the case of the two long chains molecule didodecyldimethylammonium, the spectra (Figure 9b) after 24 h in nitric acid are identical to the initial one. It confirms the importance of the length and the number of aliphatic chains linked to the nitrogen of the quaternary ammonium. It seems that at least two long aliphatic chains are stabilizing the anion exchanger in this acidic medium. Then, in [HNO_3_] = 8 mol·L^−1^ in the (11-Mercaptoundecyl)-*N*,*N*,*N*-trimethylammonium grafted monolith, the C–S bond is not destroyed—it is the quaternary ammonium which is not robust enough. Therefore, we tested the robustness of the molecule in less concentrated nitric acid. The mass spectra of the (11-Mercaptoundecyl)-*N*,*N*,*N*-trimethylammonium after 24 h in [HNO_3_] = 5 mol·L^−1^ is identical to the initial one.

Then, the versatile functionalization method is robust in concentrated HNO_3_ until 5 mol·L^−1^ and the ammonium used to functionalize the monolith needs to be composed with at least two long aliphatic chains to be resistant in stronger acidic media.

## 4. Conclusions

Centrifugal microfluidic platforms offer a great level of freedom of design along with an accurate control of flow, thanks to the centrifugal force. Centrifuge flow management was also chosen since it allowed concentrated acidic mobile phases to percolate through the stationary phase avoiding the use of any external pumping devices for radiochemistry applications. In this study, a stationary phase for separation in hard acidic medium was developed and a robust and versatile method of functionalization was proposed. The C–S bond formed via thiol-ene chemistry is strong enough to be used to graft function of interest for separation in strong nitric acid medium. The photografted anion exchanger, a quaternary ammonium, presents sufficient resistance to be used for radionuclide separation in [HNO_3_] = 5 mol·L^−1^ so the next step is its integration into the COC microsystem. Centrifugal microfluidic platforms are a promising alternative to standard procedure for chromatographic ion-exchange separations. Their use for radiochemistry applications could lead to major improvements of the analytical workflow for the nuclear industry, namely: (1) fewer manipulations and increased throughput when implemented in a glove box; (2) the reduction of solid wastes generated per analytical cycle; (3) the reduction of liquid wastes generated per analytical cycle; (4) an ease of automation and multiplexing; and (5) limited installation and maintenance costs.

## Figures and Tables

**Table 1:** Comparison of the structures of synthetized monoliths a and b and previously obtained monolith [15].

Parameter	Monolith a	Monolith b	Target Values [13]
Total porosity	56.88% ± 2.70%	62.64% ± 2.88%	86%
Globules size	2.01 ± 0.27 µm	1.02 ± 0.83 µm	1 µm
